# MetalPDB in 2018: a database of metal sites in biological macromolecular structures

**DOI:** 10.1093/nar/gkx989

**Published:** 2017-10-25

**Authors:** Valeria Putignano, Antonio Rosato, Lucia Banci, Claudia Andreini

**Affiliations:** Magnetic Resonance Center (CERM)—University of Florence, Via L. Sacconi 6, 50019 Sesto Fiorentino, Italy; Department of Chemistry—University of Florence, Via della Lastruccia 3, 50019 Sesto Fiorentino, Italy

## Abstract

MetalPDB (http://metalweb.cerm.unifi.it/) is a database providing information on metal-binding sites detected in the three-dimensional (3D) structures of biological macromolecules. MetalPDB represents such sites as 3D templates, called Minimal Functional Sites (MFSs), which describe the local environment around the metal(s) independently of the larger context of the macromolecular structure. The 2018 update of MetalPDB includes new contents and tools. A major extension is the inclusion of proteins whose structures do not contain metal ions although their sequences potentially contain a known MFS. In addition, MetalPDB now provides extensive statistical analyses addressing several aspects of general metal usage within the PDB, across protein families and in catalysis. Users can also query MetalPDB to extract statistical information on structural aspects associated with individual metals, such as preferred coordination geometries or aminoacidic environment. A further major improvement is the functional annotation of MFSs; the annotation is manually performed via a password-protected annotator interface. At present, ∼50% of all MFSs have such a functional annotation. Other noteworthy improvements are bulk query functionality, through the upload of a list of PDB identifiers, and ftp access to MetalPDB contents, allowing users to carry out in-depth analyses on their own computational infrastructure.

## INTRODUCTION

For the large majority of organisms, 30–40% of proteins require one or more metal ions to perform their biological function in cells (1;2). Additionally, metal ions play a decisive role in stabilizing the structure of nucleic acids (3). MetalPDB (4) is a resource derived from the automated analysis of all the three-dimensional (3D) structures of the adducts between biological macromolecules and metal ions or metal-containing cofactors available from the Protein Data Bank (PDB, http://www.wwpdb.org/) (5). MetalPDB stores the metal sites observed in PDB structures in the form of Minimal Functional Sites (MFSs) (6;7). Each MFS is the ensemble of atoms of the metal cofactor, the metal ligands and any other residue or chemical species within 5 Å from a ligand. The MFS describes the local 3D environment around the cofactor, independently of the larger context of the macromolecular structure in which it is embedded. The usefulness of the MFS concept has its chemico-physical foundation in the fact that the local environment of the metal has a determinant role in tuning its properties and thus its chemical reactivity. Consequently, MFSs can provide an unbiased insight into the function or mechanism of action of a metalloprotein (i.e. a protein that binds at least one metal ion or metal-containing cofactor) (6;8). The structural comparison of MFSs is useful also to predict function from 3D structure in the absence of experimental biochemical data. MetalS^3^ tool is designed to search MetalPDB for all those sites that have a similar local structure with a query site (9).

Since its first release, in 2012, MetalPDB has been widely exploited by the scientific community. In the last 12 months, there have been on average 1450 unique IPs contacting the database each month, corresponding on average to almost 4000 visits (a new visit is counted if the same IP makes requests at half-hour intervals or longer). The current release includes 287 122 sites from 50 797 structures. It was 175 115 in the first release of MetalPDB (64% growth in 6 years). MetalPDB is updated monthly in an automated manner.

In the current update of MetalPDB, we extended its contents to include various new features and expanded the information available via the web interface. A number of improvements were made to the usability of the web interface, including bulk query functionality and faster visualization of pages. As a major upgrade, we specifically addressed the identification of potential MFSs in 3D structures lacking the metal cofactor. In addition, statistical analyses on the MetalPDB contents are now available on the web site, in order to provide a better understanding of the diversity of the biochemical roles of metals.

### New contents of MetalPDB

We added secondary structure and solvent accessibility to the precomputed analyses of the structural properties of MFSs. For each metalloprotein, we used ProMotif (http://www.img.bio.uni-goettingen.de/ms-www/internal/manuals/promotif/promotif.html) (10) to calculate the secondary structure elements of the entire 3D structure and then linked this information to the MFSs within the structure. The same procedure was applied with the program NACCESS (http://wolf.bms.umist.ac.uk/naccess/) to compute the solvent accessibility of the metal-binding residues in each MFS. For the calculation of solvent accessibility, each chain in the structure was considered individually and the steric hindrance of the metal neglected.

We introduced functional annotations for MFSs. All equivalent sites (i.e. MFSs that occur at the same position within a conserved protein fold, as observed in the structural alignment of all the chains of the superfamily, and bind the same metal ions) share the same functional annotation so the clustering procedure is critical for the quality of annotation. To improve the homogeneity of groups of equivalent sites we revised our previous procedure (4) (see point 7 of the Section Database Construction) by using exclusively the Pfam domain classification (11) as the criterion to create protein superfamilies. Functional annotations are manually curated via a dedicated, password-protected annotator interface. This interface uses drop-down menus and a guided annotation procedure in order to minimize clerical errors. At the top level, we annotate the physiological relevance of each MFS by assigning it to one of these classes: ‘Physiological site’, ‘Modified Physiological site’, ‘Not physiological site’ and ‘Unknown’ (for a description, see http://metalweb.cerm.unifi.it/help/functional_annotation/). A MFS is considered to be physiological only if all the metal ions identified in the structure correspond to those required for the system to function in the cell (native metal ions), and all and only the required metals are present. In a modified physiological site, at least one metal ion has been removed, added or substituted by another metal with respect to the physiological site. A not physiological site is one that is known to be not relevant *in vivo*. When a metal ion in a structure has no donor atoms in its first coordination sphere it is automatically annotated as ‘Not physiological’; this can happen e.g. if a water molecule in the crystal structure was incorrectly assigned as a metal by the depositors. Each physiological site has one or more associated functions among ‘Catalytic’, ‘Structural’, ‘Transport’, ‘Electron transfer’, ‘Regulatory’, ‘Substrate’ and ‘Protection’ (see http://metalweb.cerm.unifi.it/help/functional_annotation/) (12). Some of these terms have a further level of annotation to improve the information content of the record. At present, a functional information is available for the majority of the sites binding iron or copper (Table 1).

**Table 1.** Percentage of annotated MFSs, grouped by metal. Data are shown only for essential metals (18)

Metal ion	Percentage of annotated sites
Cu	90%
Fe	86%
Mg	70%
Ni	35%
Mn	34%
K	32%
Na	29%
Mo	22%
Co	21%
Zn	17%
W	12%
Ca	12%
V	3%

This percentage reports on the number of MFSs with a functional annotation of any type with respect to the total number of MFSs in MetalPDB.

**Figure 1.**
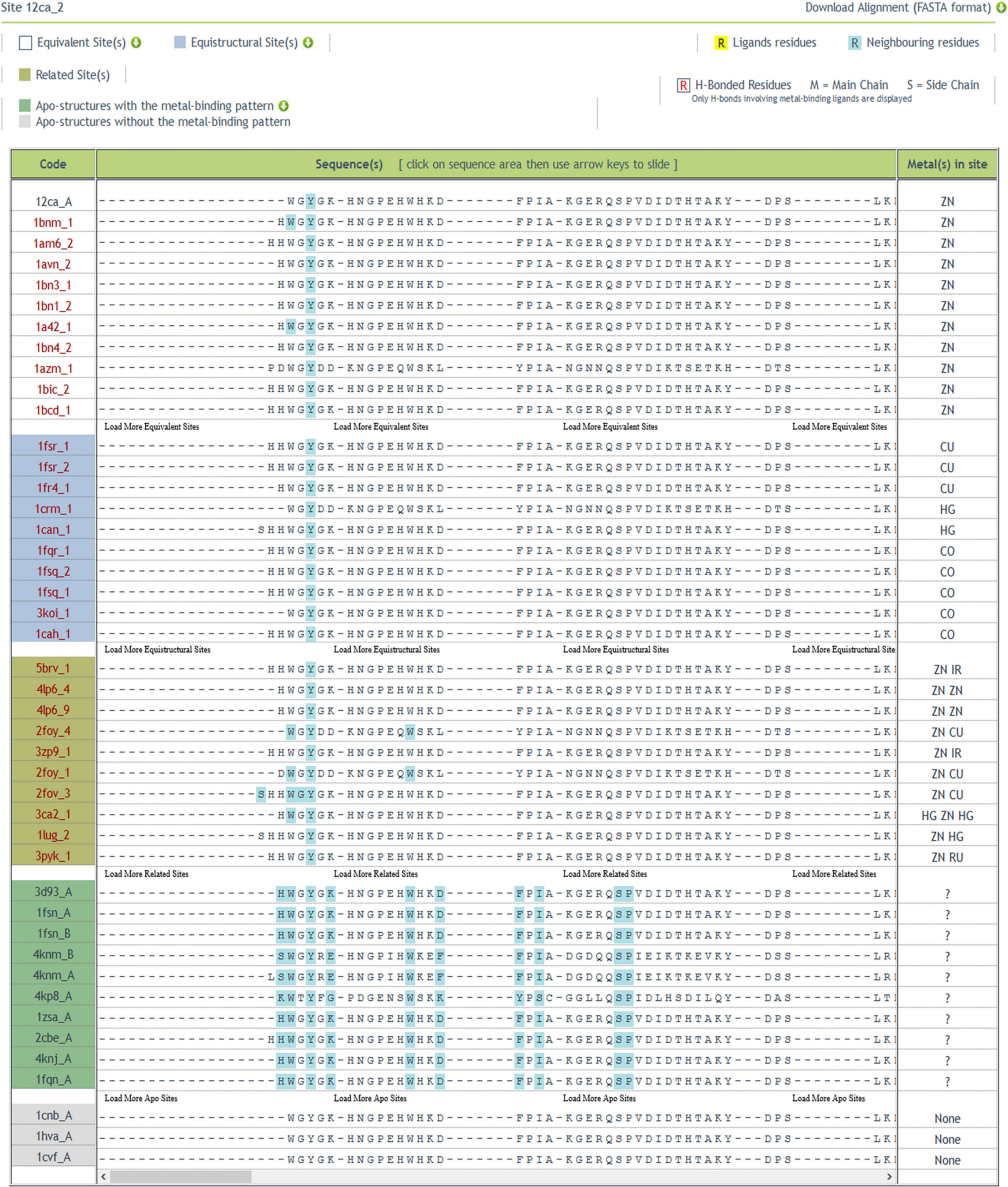
The Sequence tab for entry 12ca_2 (16). The new Sequence tab displays the sequence alignment of all proteins in the same superfamily. The proteins are grouped based on the relationship of their MFSs to the query MFS (equivalent or equistructural sites), whereas for proteins lacking any metal in the site the grouping is based on the conservation of the metal ligands (apo-structures with or without the metal-binding pattern). The metal ligands have a yellow background; the residues belonging to the MFS have a cyan background. H-bonded residues are highlighted in red.

A commonly asked question is what the structural impact of metal-binding is at the local and/or global structural level. To address this the 3D structures of the same protein with and without the cofactor needs to be compared. We therefore implemented a protocol to identify protein structures related to a structurally characterized MFS available in MetalPDB but devoid of the metal cofactor (apo-structures). To this end, we generated a multiple sequence alignment between all chains that bind equistructural MFSs (i.e. MFSs that occur at the same position within a conserved protein fold, regardless of the chemical identity of the bound metal) and the chains of apo-structures that have at least 50% identity with at least one of them. Potential MFSs in apo-structures are then identified based on the conservation of all metal-binding residues in this alignment. This procedure identifies apo-structures with the metal-binding pattern (Figure 1). Chains lacking one or more of the metal-binding residues probably have lost or significantly changed their interaction with the metal cofactor, and are listed separately as apo-structures without the metal-binding pattern (Figure 1). This provides the user with an innovative structural perspective on apo-structures, enabling the systematic analysis of the structural impact of metal binding and providing hints on the possible evolution of the MFS itself. In implementing this protocol, we realized that distinct groups of equistructural sites sometimes have some or even all metal ligands in common in the protein sequence alignment. Different groups of equistructural MFSs are created when structures with the same metal-binding protein domain have MFSs in different relative positions within the structural alignment of all the chains (4). However, the present sequence alignments reveal that this can happen while maintaining some metal ligands from the protein unchanged, i.e. the spatial shift of the MFS can be a result of structural rearrangements or flexibility rather than of evolutionary changes altering the sequence. We thus decided to dub sites that belong to different equistructural groups but share at least a protein ligand in the sequence alignment as ‘related sites’.

### The MetalPDB interface

The 2018 version of MetalPDB features an additional mode of querying the database, i.e. by providing a list of PDB identifiers. The interface analyses the list to separate entries corresponding to metal-containing, apo- or not-metal-binding structures, and then allows the user to select specific MFSs from each metal-containing entry. In this way one can select, for example, only physiologically relevant sites or only a given site in a family of metalloproteins containing multiple MFSs. After completing the selection, it is possible to create a personalized report on the properties of all the selected MFSs. For each MFS, the report can include features of the site (CATH (13)/SCOP (14)/Pfam (11)) domain containing the site, number of ligands, EC number for metalloenzymes), of the metal (coordination geometry, coordination number, metal-binding pattern) and of the ligands (donor atoms, metal-to-donor distances). The report can be downloaded as a csv file.

To facilitate the analysis of the entire MetalPDB contents, we implemented two new options for large data download: an ftp interface providing access to all the MFSs, grouped by the bound metal (each group is available as a compressed tar file), and a link to a flat file version of the database.

**Figure 2.**
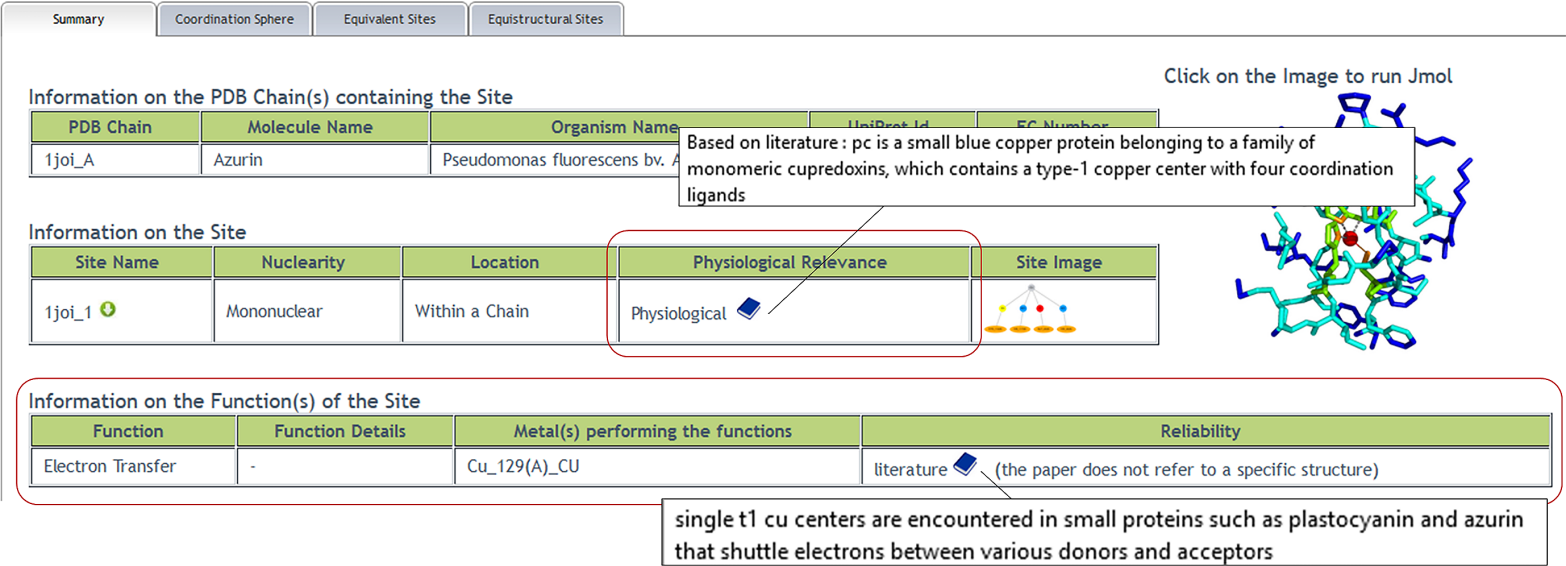
The summary page of 1joi_1 (17). The new *Information on the site* table reports the *Physiological relevance* of the site (highlighted with a red circle). Each physiological site has an associated function that is detailed in a further new table (*Information on the function(s) of the site*, also highlighted with a red circle) When an annotation is based on the literature, it is possible to display the sentence of the article that supports the functional annotation by hovering the mouse on the book icon. The book icon links to the article entry on PubMed.

MetalPDB returns results on a per-MFS basis, i.e. the result page shows the information contained in the database for an individual MFS. The information is distributed under different tabs within the page. Below we report the modified or the new tabs of the current version of MetalPDB:

- *Summary tab*: the table ‘Information on the Site’ now reports, when available, the physiological relevance of the site. When a site is ‘Physiological’, it also has an associated function, which is reported in the ‘Function Details’ table below. By hovering the mouse over the book icons, a sentence of the article supporting the annotation appears in a box (Figure 2). For Modified Physiological MFSs, we additionally provide a description of the changes with respect to the physiological site in a separate ‘Site Modification’ tab (see below).
- *Coordination Sphere tab*: each ligand is now associated with a relative solvent accessibility and with a secondary structure element.
- *Sequence tab*: this tab was not present in the previous version. It displays the sequence alignment of all the members of the protein superfamily of the query MFS (Figure 1). These include: (i) sequences harbouring equivalent sites (white) and (ii) sequences harbouring equistructural sites (blue); (iii) sequences with ‘related sites’ (light green), (iv) sequences of apo-structures that conserve all the metal-binding residues of the query MFS (dark green) and (v) sequences of apo-structures which have lost at least one metal-binding ligand with respect to the query MFS (grey). A structural superposition of the putative sites in the apo-structures with the metal-binding pattern to the query MFS can be downloaded. It is also possible to download the alignment of all the sequences. The user can move along the alignment by shifting it right or left to inspect its different regions, or by showing more or less sequences. A color code highlights the protein residues forming the MFS as well as the position and interactions of the metal-binding residues.
- *Site Modification tab:* this new tab is present when the query MFS is annotated as a ‘Modified Physiological Site’. It details the modifications of the query MFS with respect to the physiologically relevant site(s).

### Statistics pages

**Figure 3.**
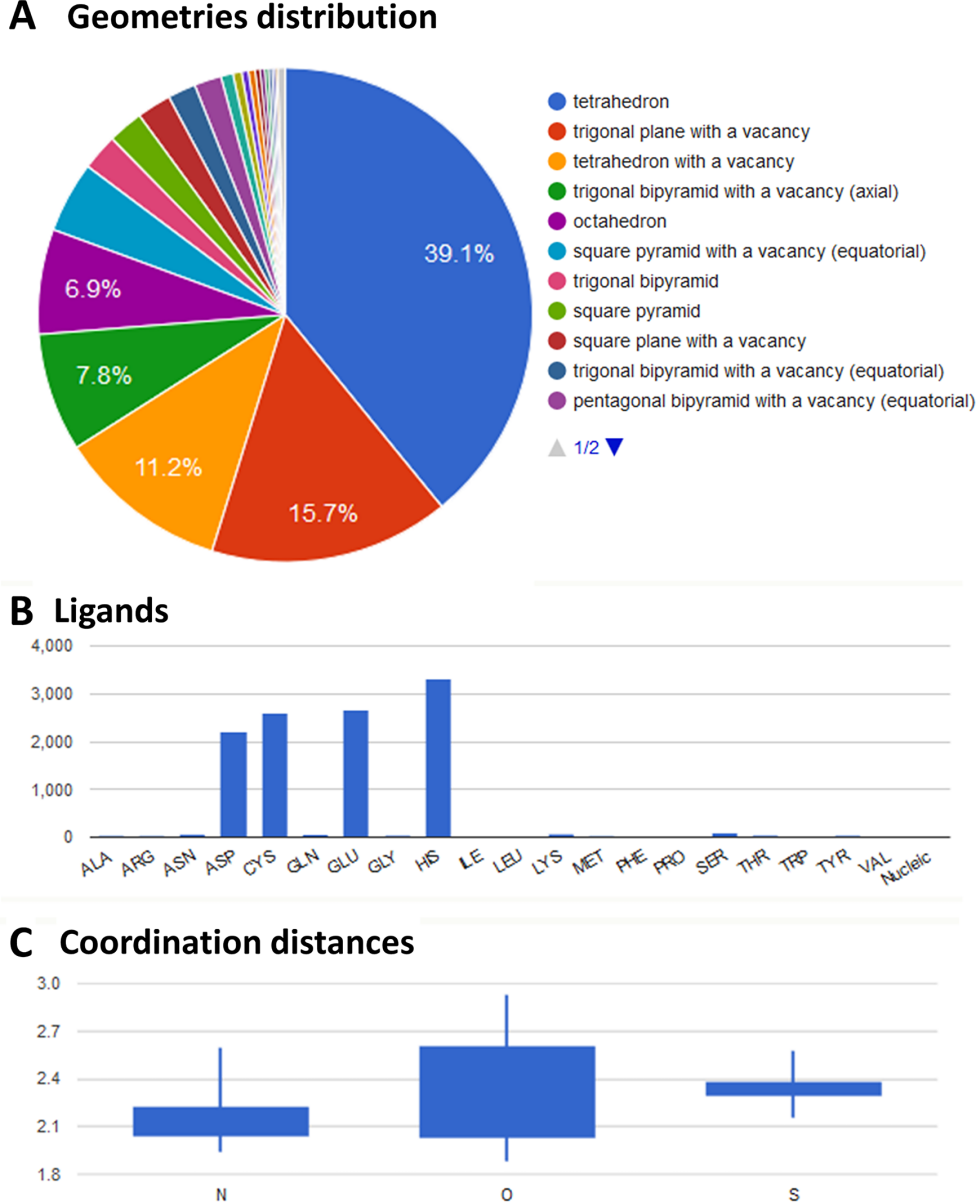
Example of statistics for zinc coordination spheres in the PDB. The information is accessible from the ‘Per metal’ statistics menu. (**A**) Pie chart displaying the coordination geometries of zinc sites; (**B**) histogram reporting the occurrence of residues in the first coordination sphere of zinc ions; (**C**) distances between zinc ions and different donor atoms.

We have extended the previous version of MetalPDB to provide extensive statistics on its contents, providing both structural and functional information. Several different pages, which can be accessed via the *Statistics* drop-down menu of the navigation bar, are available:

- *Summary*, which lists the number of sites, atoms and PDB structures contained in MetalPDB on a per-metal basis;
- *Metals in PDB*, which provides an overview of the fractional occurrence of metal-binding structures in different repositories or for different macromolecule types, a histogram of the number of MFS with a given nuclearity (number of metal ions per site), and a statistics of the most common coordination geometries observed in MetalPDB;
- *Per Geometry*, which provides statistics per each coordination geometry defined in FindGeo (15). By clicking on the geometry of interest, the user enters a page describing which metals were assigned that geometry in MetalPDB and how many different metal-binding patterns adopted that geometry for each metal;
- *Metal domains*, which provides an overview of the fractional occurrence of metal-binding domains in domain databases, in total and on a per-metal basis. For the SCOP and CATH databases, the per-metal statistics is further subdivided by domain class;
- *Per metal*, which enables two different kinds of analyses: coordination geometries or metal ligand distributions. In this page, the users selects one specific metal ion for which s/he wants to obtain statistics; then the desired analysis is selected by pressing a button at the bottom of the page. In the Geometries section, MetalPDB reports the occurrence of all regular coordination geometries (Figure 3A), the distribution of aminoacidic ligands for each geometry, and the number of different metal-binding patterns observed for the selected metal as a function of the coordination geometry. In the Ligands section, MetalPDB reports the statistics on the presence of aminoacidic or nucleic ligands in the coordination sphere of the selected metal (Figure 3B), the distribution of metal to donor atom distances (Figure 3C), and data on non-bonded interactions between aminoacidic ligands and other aminoacids of the protein (so-called second-sphere interactions);
- *Metals in enzymes*, which reports on the presence of metal sites in enzymes as well as on the occurrence of the different metal ions among the six EC classes and on the distribution of the six EC classes among metalloproteins on a per-metal basis (note that we include both catalytic and non-catalytic MFSs for any protein that has a EC number associated);
- *Metal substitutions in sites*, which reports on the distribution of the different metal ions replacing any given metal in all the sites (for example showing that the most common replacement for Ni is Zn, whereas for Mg it is Ca); this statistics is derived from the comparison of equistructural groups.

All these pages are updated every time the database content is updated to the newest PDB release. Several of the statistics listed above, in particular those involving ligands and metal-binding patterns, address only metalloproteins.

## CONCLUSIONS AND PERSPECTIVES

The number of structurally characterized metal-binding sites in biological macromolecules is still experiencing a significant growth. We have coped with this growth (64% in 6 years) by reviewing and improving the protocols for the construction of MetalPDB contents. In parallel, we expanded the options available to users for interacting with MetalPDB as well as the amount and complexity of precomputed structural and functional information displayed in the pages of MetalPDB. In the next releases of MetalPDB, we will continue to improve the functional information, also by enabling queries and statistics that target functional aspects directly. An important advancement is the functional annotation of individual MFSs, which is only partial at present. In the future development of MetalPDB we will work on increasing the coverage of annotated MFSs.
